# Allogeneic stem cell transplantation combined with conditioning regimen including donor-derived CAR-T cells for refractory/relapsed B-cell lymphoma

**DOI:** 10.1038/s41409-022-01903-3

**Published:** 2022-12-22

**Authors:** Fan Yang, Hui Shi, Teng Xu, Rui Liu, Yang Lei, Ruiting Li, Biping Deng, Tong Wu, Xiaoyan Ke, Kai Hu

**Affiliations:** 1 Department of Adult Lymphoma, Beijing Boren Hospital, Beijing, China; 2 Cytology Laboratory, Beijing Boren Hospital, Beijing, China; 3 Department of Bone Marrow Transplantation, Beijing Boren Hospital, Beijing, China

**Keywords:** Haematopoietic stem cells, Immunology

## To the Editor:

In recent years, immunochemotherapy and targeted therapies have significantly improved patient survival [1], whereas the proportion of patients with refractory/relapsed B-NHL (R/R B- NHL) was found to be lower than 50%, with extremely poor survival [2–4]. In 2017, the United States Food and Drug Administration approved the administration of chimeric antigen receptor (CAR) T-cell therapy targeting CD19 antigen for the third or later line of systemic therapy for patients with R/R B-NHL [5], whereas ~20–30% of patients still failed to respond or had relapses.

Allogeneic hematopoietic stem cell transplantation (allo-HSCT) has been presented for multi-line therapy of R/R B-NHL patients [6], whereas the long-term survival rate in patients with high tumor burden (HTB) before transplantation was only ~4% [7, 8]. To explore appropriate therapeutic options for patients with R/R B-NHL, CAR-T cells from the same donor (donor- derived CAR-T) were involved in the conditioning of allo-HSCT, utilizing the immune effects to reduce the HTB before transplantation. This procedure was conducted to decrease the high relapse rate and to improve survival.

From September 2020 to May 2021, 8 eligible adult patients with R/R B-NHL were enrolled, including high-grade B-cell lymphoma (*n* = 4), diffuse large B-cell lymphoma, NOS (*n* = 3), and Richter transformation (*n* = 1). Based on the 5th edition of the World Health Organization Classification of Haematolymphoid Tumors: Lymphoid Neoplasms. Patients aged 18–60 years, with Eastern Cooperative Oncology Group status 0–2 and no uncontrollable infections, intracranial hypertension, or organ failure (Table S1 and S2). Patients were required to give written informed consent. The protocol was approved by the Institutional Review Board of Beijing Boren Hospital (ChiCTR2000040665).To be eligible for participation in this study, an individual must meet the criteria described in the supplemental materials.

Peripheral blood mononuclear cells were isolated from the haplotype donors for allo-HSCT, and CD3 + T lymphocytes were separated by using antigencoated immunomagnetic beads. CD19/CD20/CD22 expression in tumor tissues was identifed by IHC and FCM, which was the basis for selecting targets for CAR T-cells. Patients received allo-HSCT in combination with donor-derived CAR T-cell therapy with the T/Bu/Flu (thiotepa 5 mg/kg·d, -16d~-15d;busulfan 3.2 mg/kg·d, -16d~-14d;fludarabine 30 mg/m2·d, -16d~-12d)-based conditioning regimen. Te detailed dosages were adjusted according to the fundamental status and tolerance of the patients. Taking the date of hematopoietic stem cells transfusion as day 0, donor-derived CAR T-cell was transfused on day-9 and -8. Tacrolimus, antithymocyte globulin and a short course of MTX and mycophenolate mofetil were used to prevent graft-versus-host disease (GVHD) (Fig. S1).

Multicolor fow cytometer and quantitative polymerase chain reaction (qPCR) were used to detect the CAR T-cell concentration in the blood. Enzyme-linked immunosorbent assay was used to dynamically monitor the peripheral serum cytokines. The criteria for cytokine release syndrome (CRS), neurotoxicity, GVHD, and tumor assessment are listed in the supplemental materials. Overall survival (OS) was defined as the time from the date of allo-HSCT to the censoring date of follow-up examination or death. Progression-free survival (PFS) was defined as the time from the date of allo-HSCT to disease relapse or the censoring date of the follow-up examination.

The *t*-test or Wilcoxon test was implemented to compare the CAR-T cell expansion and percentage of different lymphocyte subtypes. The Kaplan–Meier method was employed to estimate the probability of OS and EFS.

The results obtained in this study have been represented in Table 1.

**Table 1:** Treatment efficacy and survival of patients.

Patient	Disease status before transplantation	Efficacy 2 months after CAR-T cells	Efficacy 5 months after CAR-T cells	Time to disease progression	Current efficacy	Survival state	Time of death/follow up duration	Cause of death
1	PD	PD				Death	149 d	Disease progression
2	PD	PR	PD			Death	220 d	Disease progression
3	SD	CR		134 d after transplantation		Death	171 d	Disease progression
4	PR	CR	CR			Death	294 d	Infection
5	PR	PR	CR		CR	Survival	247 d	
6	PR	PR	CR		CR	Survival	242 d	
7	PR	PR	CR		CR	Survival	162 d	
8	PR	PR	CR		CR	Survival	156 d	

## Disease status after cytoreduction before transplantation

Eight eligible patients were enrolled, all of whom had failed autologous anti-CD19 CAR- T therapy (Table S1). After the bridging therapy (including chemotherapy and targeted therapy), 5, 1, and 2 patients had partial remission (PR), stable disease (SD), and PD, respectively.

## Donor-derived CAR T-cell expansion and persistence

The median dose of donor-derived CAR-T infusion was 4 (range, 0.78–4.88) × 10^6^/kg.

FCM detected the expansion of donor-derived CAR-T cell in the peripheral blood of all 8 patients.

Donor-derived CAR-T cells peaked at a median of 10 (range, 7–35) days with a median *percentage* of peak CAR vector transgene copies(CAR vector transgene copies/Human single copy gene from PB mononuclear cells) 8.0162% (range, 0.09–116.8%) by qPCR in blood. The results of statistical analysis showed that the dose of infusion of donor-derived CAR- T was not significantly correlated with the peak value of in vivo expansion of CAR-T cells (*P* = 0.09). The peak donor-derived CAR-T was higher in patients in the PR condition than in SD/PD before allo-HSCT (*P* < 0.0001) (Figs. S2 and S3).

## Safety assessment

CRS occurred in all eight patients (100%), including grades I, II, and III CRS in 4 (50%), 1 (12.5%), and 3 (37.5%) patients (Fig. S4), respectively. The increased levels of interferon γ and interleukin-6 were associated with the grade of CRS (Fig. S5). No neurotoxicity was found. Three patients with CRS grade III cured after methylprednisolone application. Seven days after transfusion of donor-derived CAR T-cells, granulocyte colony-stimulating factor–mobilized peripheral blood stem cells (G-PBSCs) were infused. The median number of mononuclear cells was 5.2 (range, 4.18–8.24) × 10^8^/kg. The median numbers of CD34 + and CD3 + cells were 6.0(range, 3.00–8.19) × 10^6^/kg and 2.37 (range, 1.66–3.75) × 10^8^/kg (Table S2). The engraftments of the absolute neutrophil granulocytes exceeding 0.5 × 10^9^/L and platelets exceeding 20 × 10^9^/L were achieved on median of days 15 (range, 11–22) and 16 (range, 14–85), respectively. Full-donor chimerism was achieved on day +28 by analysis of short tandem repeats after transplantation.

Two patients (25%) developed acute GVHD (both grades II of skin), and two patients (25%) developed chronic GVHD (mild oral ulceration and skin detachment). Six (75%) patients had cytomegalovirus viremia. Three patients (37.5%) developed grade I hemorrhagic cystitis BK. Viral cystitis was found in two patients (25%) and early intestinal infection and dysbacteriosis in five patients (62.5%).

## Efficacy and survival

At a median follow-up period of 249 (range 179–263) days, complete remission (CR) was achieved in six cases (6/8, 75%), although one patient achieved CR and then relapsed. The median OS and PFS was 294 (95% CI: 149, 294) days, with a 6-month OS and PFS of 75% and 62.5%, respectively. There were statistically significant differences in OS (*p* = 0.0083) and PFS (*p* = 0.0042) between the PR and SD /PD groups prior to transplantation. Three cases (3/8, 37.5%) died from disease progression with pre-transplant disease status SD and PD, one patient (1/8,12.5%) died from infection (Fig. S6).

Theoretically, donor-derived lymphocytes overcame the failure of autologous CAR-T cell treatment caused by the malfunction of autologous lymphocytes. However, the obstacles for unmodified allogeneic CAR-T cells are poor in vivo expansion and persistence due to alloreactivity, failing to achieve the goal of reducing tumor burden [9]. Therefore, the conditioning regimen by allo-HSCT proposed in this study could simultaneously achieve two goals: firstly, the intensity of conditioning disrupted the patients’ immunity defense and eliminated the lymphocytes, ensuring the expansion of donor-derived CAR-T, and the immunological effects further reduced the tumor burden before transplantation; secondly, the consequent transfusion of hematopoietic stem cells allowed for hematopoiesis and immunological reconstruction, and the donor-derived CAR-T cells from the same immune platform survived and exerted oncological surveillance effects.

In general, we found that allo-HSCT combined with conditioning regimen including donor-derived CAR-T cells are safe. CRS was manageable, and no neurotoxicity occurred. Furthermore, the incidence of aGVHD did not increase post-transplantation, and transplant-related complications were also under control.

In addition, further analysis showed that PFS (6-month PFS 62.5%) was longer in our study than in previous studies [8] (1-year PFS 29–44%). We speculated that the observed differences might be due to the following: firstly donor-derived CAR T cells expanded successfully and persistence at low doses in the body possibly reduced tumor burden and led to remission in patients; However, testing efficacy after infusion of donor CAR-T cells was difficult because of the myelosuppressed state of the patient in the transplant ward and the high risk of infection. Therefore, it was not possible to accurately assess the contribution of donor CAR -T cells to reducing tumor burden. Secondly immunosuppression was not increased to prevent GVHD, according to previous reports, so the allogeneic graft-versus-lymphoma effect probably also played a role in reducing tumor burden [10]. However, patients with pre-transplant status SD or PD could not benefit from this treatment. Therefore, a long-term follow-up period is required.

This study was registered in the Chinese Clinical Trial Registry/International Clinical Trial Registry Platform of WHO (ClinicalTrials#: ChiCTR2000040665).

## Supplementary information

SUPPLEMENTAL MATERIAL

SUPPLEMENTAL MATERIAL

SUPPLEMENTAL MATERIAL

SUPPLEMENTAL MATERIAL

## Data Availability

The datasets used and/or analyzed during the current study are available from the corresponding author on reasonable request.

## References

[1] Pfreundschuh M, Kuhnt E, Trümper L, Osterborg A, Trneny M, Shepherd L (2011). CHOP-like chemotherapy with or without rituximab in young patients with good-prognosis diffuse large-B-cell lymphoma: 6-year results of an open-label randomised study of the MabThera International Trial (MInT) Group. Lancet Oncol.

[2] Tsai T, Goodman S, Saez R, Schiller G, Adkins D, Callander N (1997). Allogeneic bone marrow transplantation in patients who relapse after autologous transplantation. Bone Marrow Transpl.

[3] Cheah CY, Fowler NH, Wang ML (2016). Breakthrough therapies in B-cell non-Hodgkin lymphoma. Ann Oncol.

[4] Epperla N, Hamadani M (2017). Hematopoietic cell transplantation for diffuse large B-cell and follicular lymphoma: Current controversies and advances. Hematol Oncol Stem Cell Ther.

[5] Makita S, Imaizumi K, Kurosawa S, Tobinai K (2019). Chimeric antigen receptor T-cell therapy for B-cell non-Hodgkin lymphoma: opportunities and challenges. Drugs Context.

[6] Corradini P, Tarella C, Olivieri A, Gianni AM, Voena C, Zallio F (2002). Reduced-intensity conditioning followed by allografting of hematopoietic cells can produce clinical and molecular remissions in patients with poor-risk hematologic malignancies. Blood.

[7] Izumi K, Kanda J, Nishikori M, Arai Y, Ishikawa T, Yoshioka S (2019). Outcomes of allogeneic stem cell transplantation for DLBCL: a multi-center study from the Kyoto Stem Cell Transplantation Group. Ann Hematol.

[8] Fenske TS, Hamadani M, Cohen JB, Costa LJ, Kahl BS, Evens AM (2016). Allogeneic Hematopoietic Cell Transplantation as Curative Therapy for Patients with Non-Hodgkin Lymphoma: Increasingly Successful Application to Older Patients. Biol Blood Marrow Transpl.

[9] Depil S, Duchateau P, Grupp SA, Mufti G, Poirot L (2020). ‘Off-the-shelf’ allogeneic CAR T cells: development and challenges. Nat Rev Drug Disco.

[10] Chen XH, Zhang C, Zhang X, Gao L, Gao L, Kong PY (2009). Role of antithymocyte globulin and granulocyte-colony stimulating factor-mobilized bone marrow in allogeneic transplantation for patients with hematologic malignancies. Biol Blood Marrow Transpl.

