# Supplementary material for: Allogeneic stem cell transplantation combined with conditioning regimen including donor-derived CAR-T cells for refractory/relapsed B-cell lymphoma
Source: Bone Marrow Transplant. 2022 Dec 22;58(4):440–2. doi: 10.1038/s41409-022-01903-3 (PMC10073016; doi:10.1038/s41409-022-01903-3)
Supplement: Supplementary file 1 — SUPPLEMENTAL MATERIAL [file 41409_2022_1903_MOESM1_ESM.docx]

**Supplementary materials**

- **Conditioning regimen for Allo-HSCT：**

Thiotepa 5mg/kg•d -16d~-15d

Busulfan 3.2mg/kg•d -16d~-14d

Fludarabine 30mg/m^2^•d -16d~-12d

- **Regimen of preventing graft-versus-host disease (GvHD) ：**

Continuous pumping of tacrolimuss(FK506) (0.02 mg/kg/d) for 24 h was conducted since the day of transfusion of hematopoietic stem cells. The dose was gradually reduced until discontinuation according to the disease conditions after hematopoietic reconstitution. Mycophenolate mofetil(MMF) (0.5 g, Q12h) was administered, the dose was reduced to 0.25 g (Q12h) after engraftment of neutrophils, and the treatment was discontinued on 28th d. Methotrexate (MTX) injection was performed on the 1st d with the dose of 15 mg/m^2^ (body surface area), and on 3rd, 6th, and 11th d with the dose of 10 mg/m^2^ (body surface area). Anti-human Thymocyte Globulin(Rabbit) (ATG)with the dose of 2.5 mg/kg/d was administered on 14th and 15th d. The treatment regimen could be adjusted according to the other conditions of patients after approval by the prominent investigator.

- **CAR-T product details：**

The CAR vectors used to prepare CAR-T cells are both lentiviral vectors carrying a second generation CAR with 4-1BB co-stimulatory and CD3ζ signaling domains. The antigen recognition domains of CD19,CD22 and CD20 specific CARs are single chain variable fragments (scFvs) obtained from a human antibody phage display library.Donor-derived cells were collected for producing CAR-T cells, which were transfected by lentiviral vectors and cultured for 5-8 days.

(CAR vectors in this study have been described before and may refer to references.^S1^

- In order to be eligible to participate in this study, an individual must meet all of the following criteria:

**1、Inclusion criteria**

1) Patients who have been diagnosed with B-cell lymphoma and have relapsed after treatment with second-line or higher regimens (standard treatment including Rituxan and high-dose chemotherapy, or half a year after autologous hematopoietic stem cell transplantation), autologous CART cell therapy is invalid or the disease has progressed; 2) Male or female patients, aged 18-60 years (including 18 years old); 3) No severe allergies; 4) Physical fitness score 0-2 points; 5) The estimated survival period >=90 days; 6) Tumor tissue immunohistochemistry are positive; 7) The donor can collect enough lymphocytes and hematopoietic stem cells; 8) The patient voluntarily signed an informed consent form.

**2、Exclusion criteria：**

1) Intracranial hypertension or brain consciousness disorder; 2) Symptomatic heart failure or severe arrhythmia; 3) Symptoms of severe respiratory failure; 4) With other types of malignant tumors; 5) Diffuse intravascular coagulation; 6) Serum creatinine and/or urea nitrogen >=1.5 ULN; 7) Suffer from sepsis or other infections that are difficult to control; 8) Suffer from uncontrollable diabetes; 9) Severe mental disorder; 10) There are obvious intracranial lesions in head MRI examination; 11) Have received organ transplantation (excluding autologous hematopoietic stem cell transplantation); 12) Female patients (patients with fertility) have a positive blood HCG test; 13) Active hepatitis (including positive copy number of hepatitis B DNA and hepatitis C RNA) and positive screening for AIDS and syphilis; 14) DSA (anti-donor specific antibody) is positive.

- **Diagnostic criteria**

1、Cytokine release syndrome (CRS) and neurotoxicity was assessed according to the Penn grading scale, and the Common Terminology Criteria for Adverse Events (CTCAE; Ver. 5.0).^S2^

2、GVHD was assessed according to Graft versus Host Disease (GVHD) International Alliance (MAGIC) Grading Standard.^S3^

3、 The laboratory monitoring was done on d0, d3, d7,d14, d21, and d28 and then monthly until 6 months after transfusion of CART. Tereafter, the monitoring was further continued every 3 months until 24 months after the transfusion.

4、The first PET-CT assessment of efficacy was conducted 2 months after allo-CAR-T cell transfusion, followed by every 3 months for 2 years after the transfusion. Tumor response assessments were performed locally per Lugano 2014 classification.

- **Detail of aGVHD**

*Patients #5 was found with grade II aGvHD (skin) on day 26 after transplantation. Rash scattered over head and neck, forehead, back and abdomen, with itching, covering 35% of the area.Rash disappears within 2 weeks after treatment with intermittent low-dose MTX(10mg/m^2^，PO，once a week, twice in total).**Patients #7 was found with grade II aGvHD (skin) on day 24 after transplantation.Rash scattered over forehead and abdomen, without itching, covering 40% of the area.Rash disappears within 20 days after treatment with intermittent low-dose MTX(10mg/m2，PO，once a week, three times in total).No glucocorticoids were used in either patient.

**References**

**S1.**Pan J, Niu Q, Deng B, et al. CD22 CAR T-cell therapy in refractory or relapsed B acute

lymphoblastic leukemia. Leukemia,2019;33:2854-2866.doi: 10.1038/s41375-019-0488-7.

**S2.**A. Lee DW, Santomasso BD, Locke FL, et al: ASTCT consensus grading for cytokine release syndrome and neurologic toxicity associated with immune effector cells. Biol Blood Marrow Transplant,2019,25:625-638, .doi: 10.1016/j.bbmt.2018.12.758.

**S3.**B.Schoemans HM, Lee SJ, Ferrara JL, et al. EBMT-NIH-CIBMTR Task Force position

statement on standardized terminology &guidance for graft- versus- host disease assessment. Bone Marrow Transplant, 2018, 53 (11):1401- 1415.doi: 10.1038/s41409-018-0204-7.
