# Supplementary material for: Allogeneic stem cell transplantation combined with conditioning regimen including donor-derived CAR-T cells for refractory/relapsed B-cell lymphoma
Source: Bone Marrow Transplant. 2022 Dec 22;58(4):440–2. doi: 10.1038/s41409-022-01903-3 (PMC10073016; doi:10.1038/s41409-022-01903-3)
Supplement: Supplementary file 2 — SUPPLEMENTAL MATERIAL [file 41409_2022_1903_MOESM2_ESM.pptx]

## Slide 1
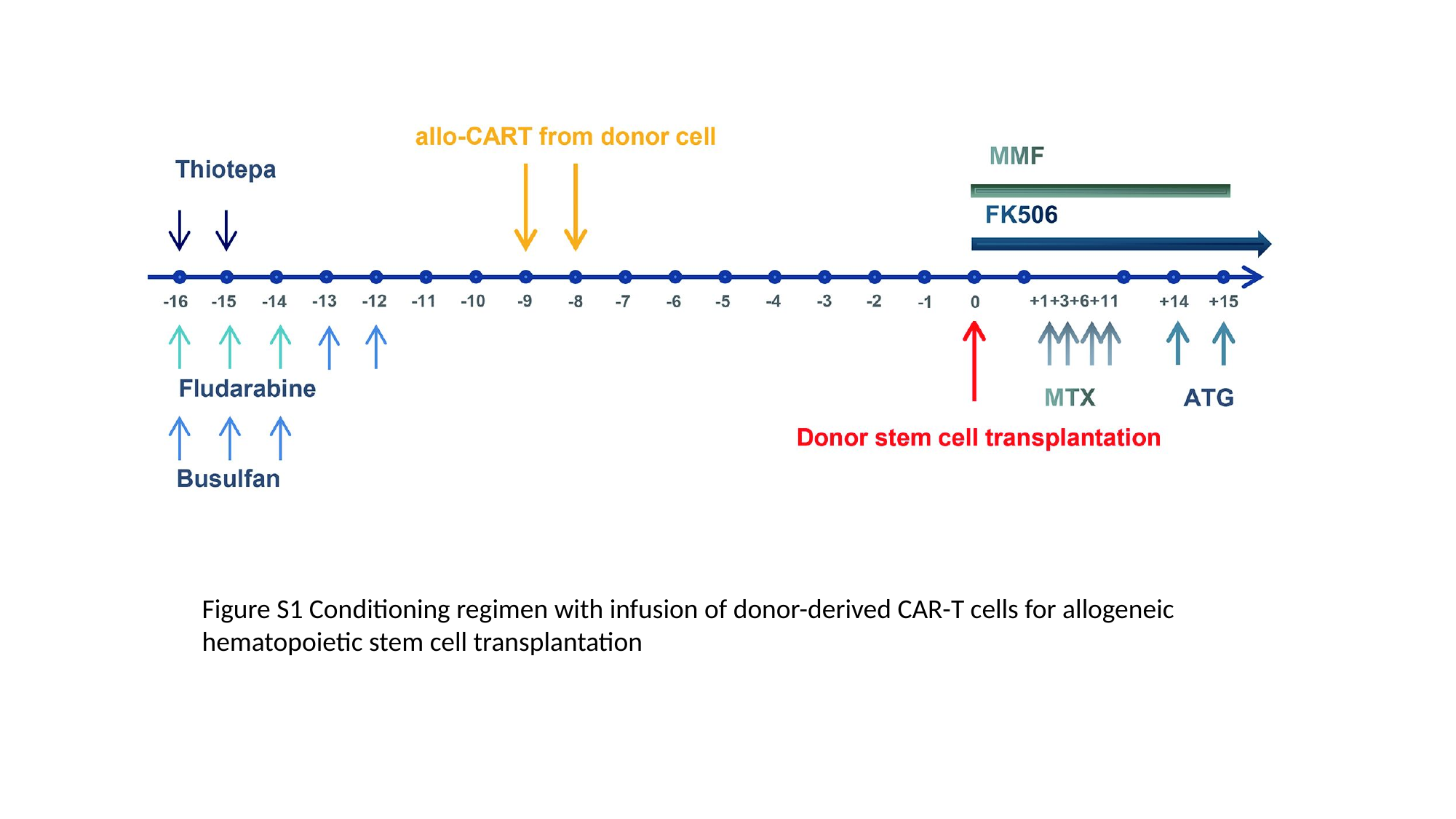

Figure S1 Conditioning regimen with infusion of donor-derived CAR-T cells for allogeneic hematopoietic stem cell transplantation

## Slide 2
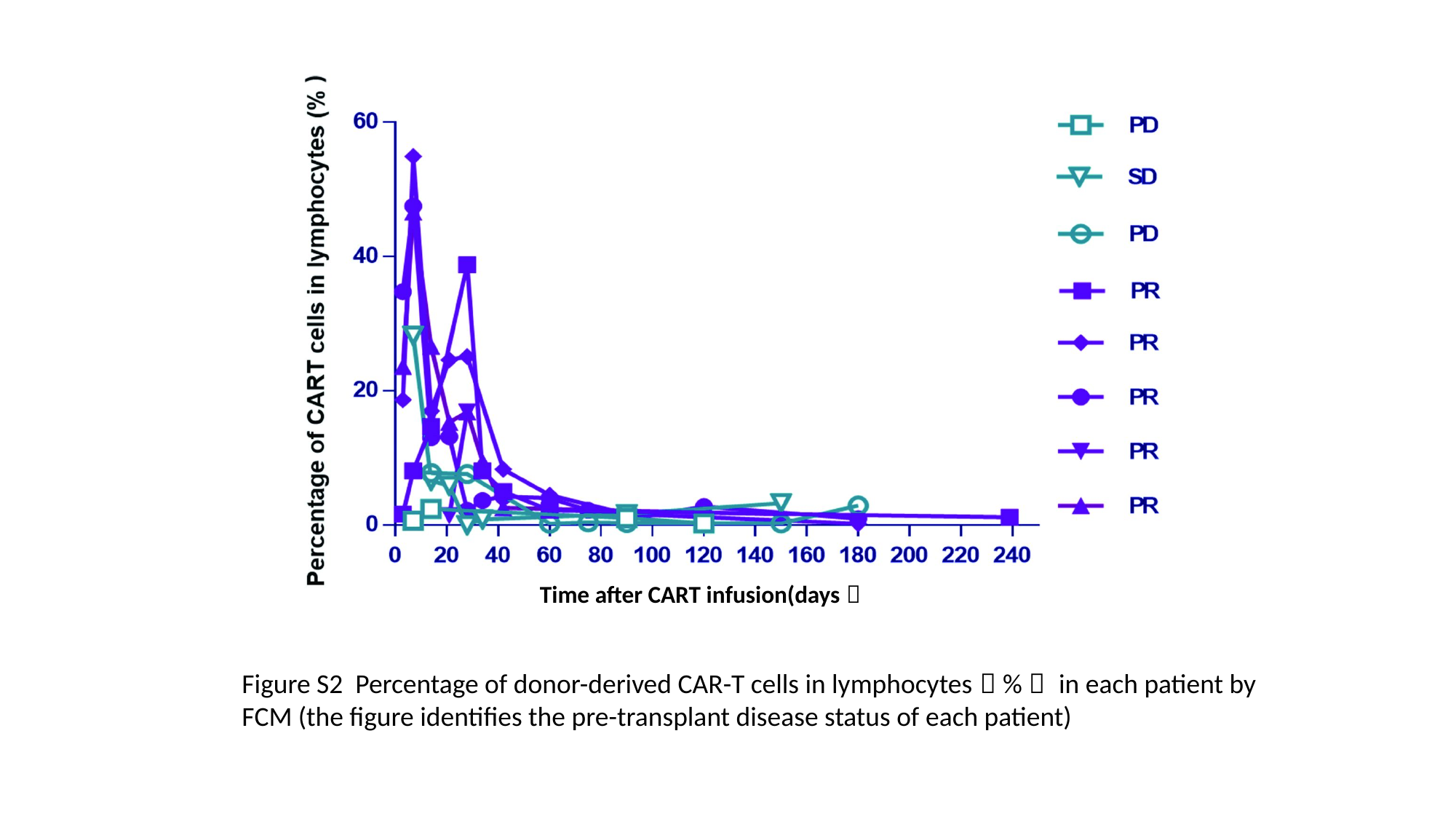

Time after CART infusion(days）
Figure S2 Percentage of donor-derived CAR-T cells in lymphocytes（%） in each patient by FCM (the figure identifies the pre-transplant disease status of each patient)

## Slide 3
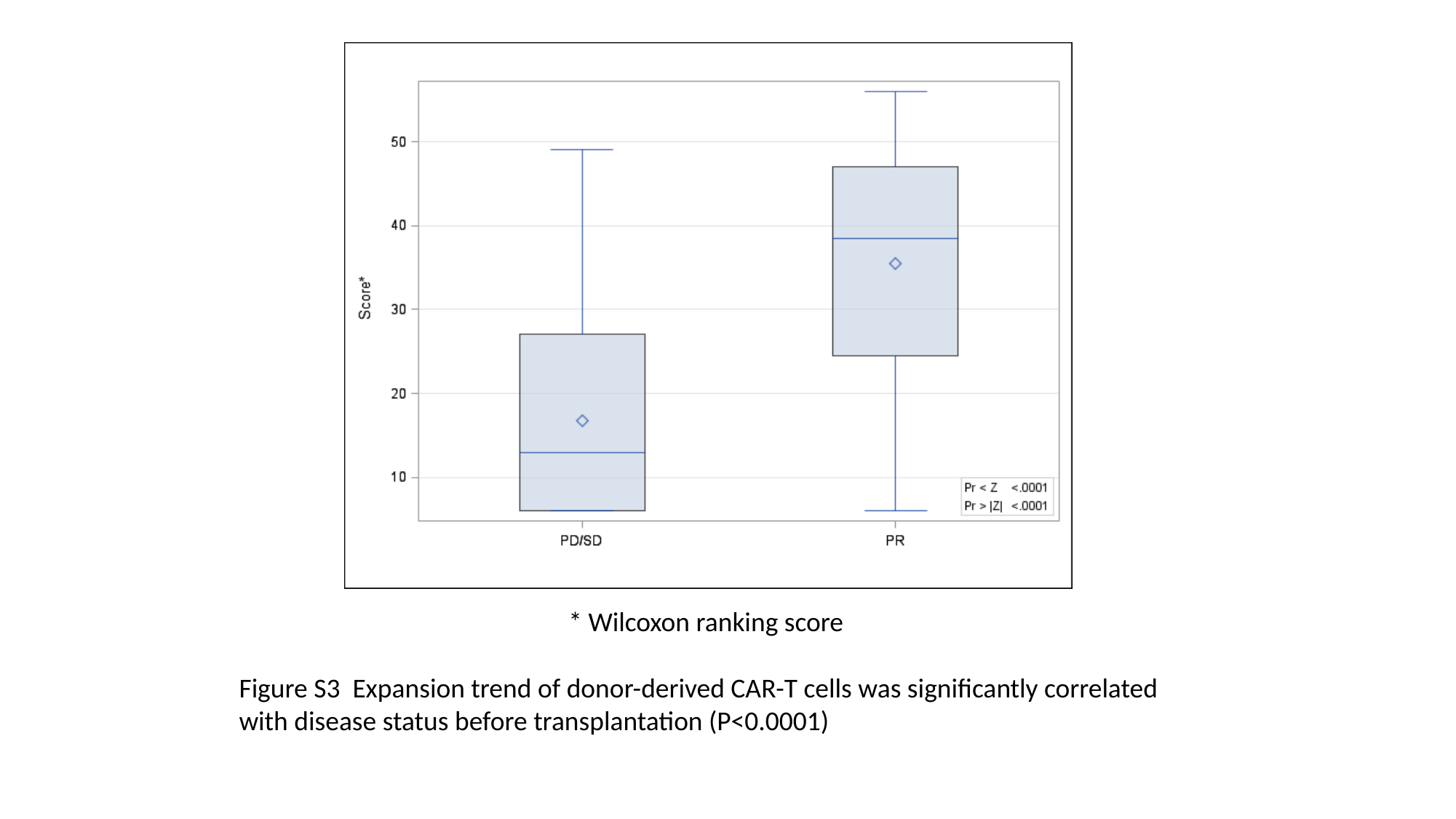

* Wilcoxon ranking score
Figure S3 Expansion trend of donor-derived CAR-T cells was significantly correlated
with disease status before transplantation (P<0.0001)

## Slide 4
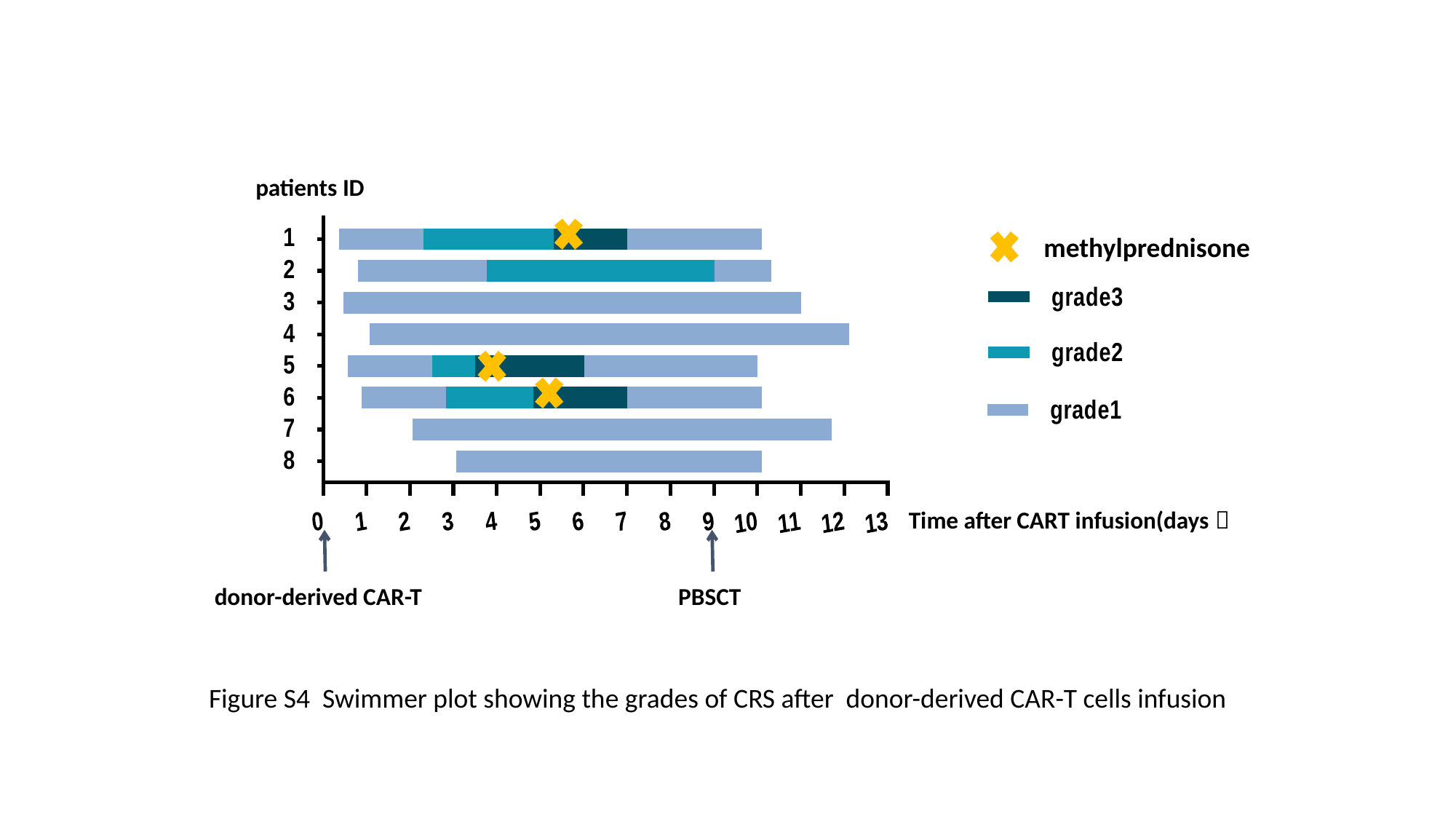

methylprednisone
 donor-derived CAR-T PBSCT
patients ID
Time after CART infusion(days）
Figure S4 Swimmer plot showing the grades of CRS after donor-derived CAR-T cells infusion

## Slide 5
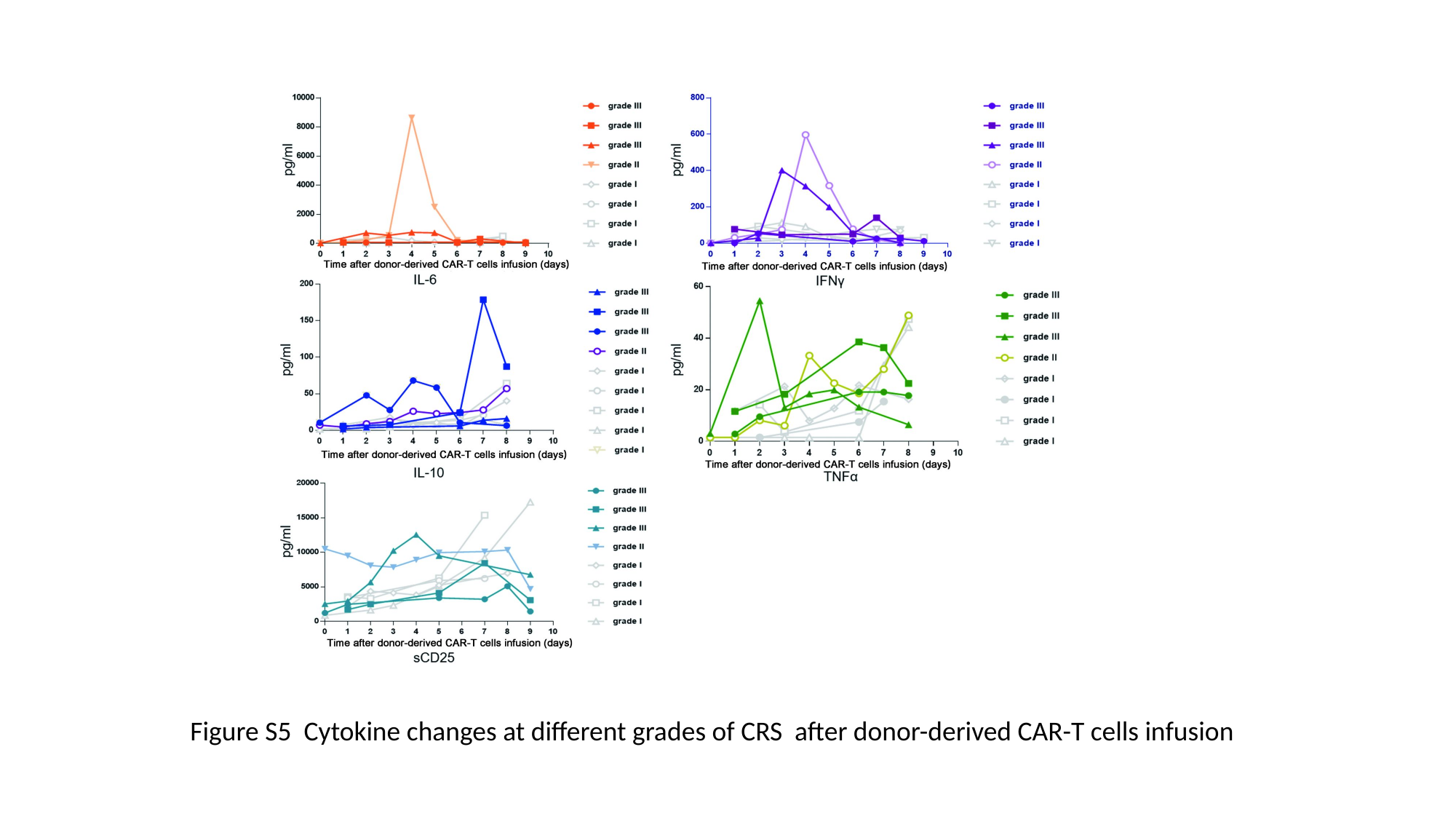

Figure S5 Cytokine changes at different grades of CRS after donor-derived CAR-T cells infusion

## Slide 6
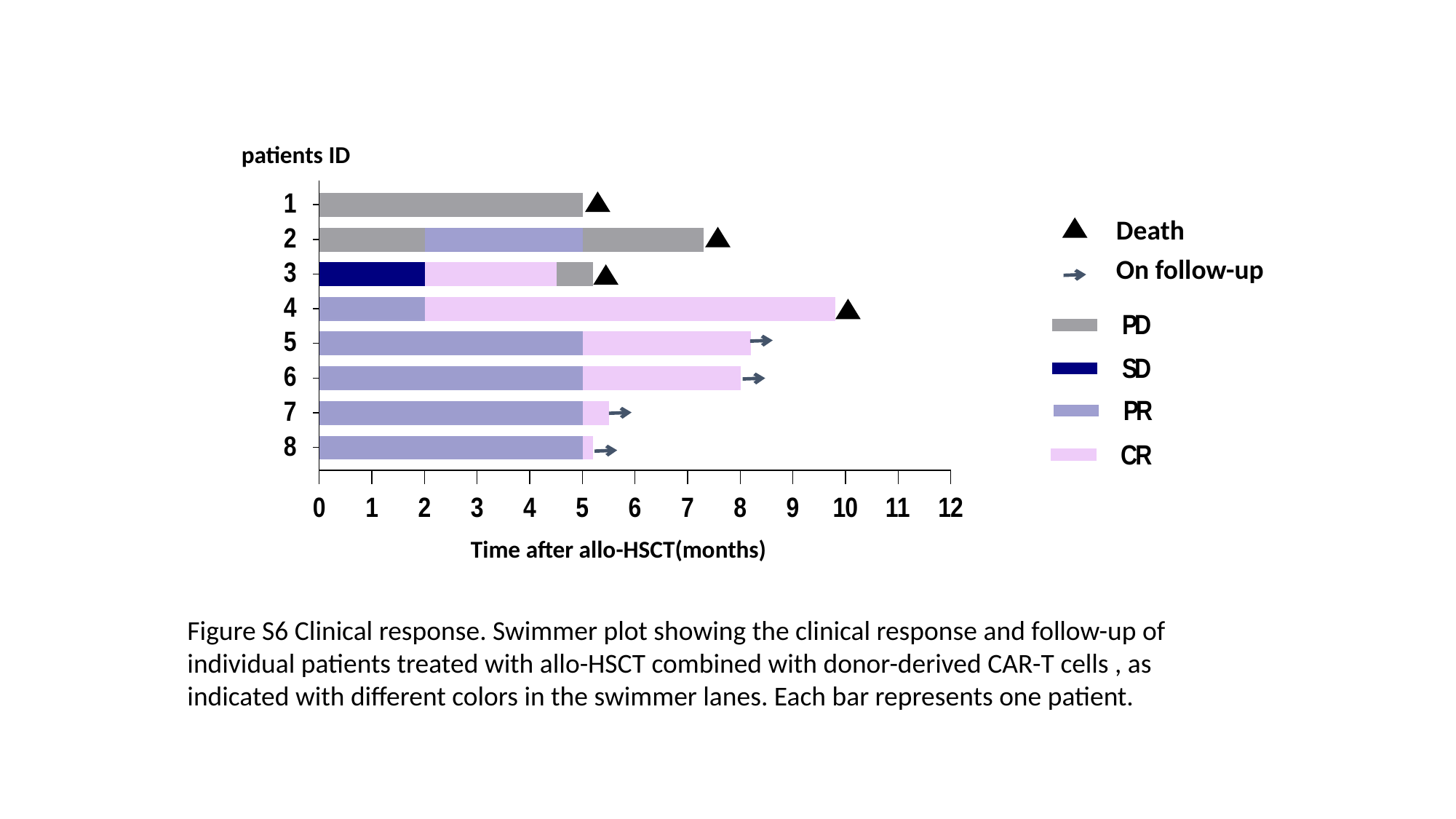

patients ID
Death
On follow-up
Time after allo-HSCT(months)
Figure S6 Clinical response. Swimmer plot showing the clinical response and follow-up of individual patients treated with allo-HSCT combined with donor-derived CAR-T cells , as indicated with different colors in the swimmer lanes. Each bar represents one patient.
